# Changes in Corneal Morphology and Biomechanics in Cases of Small Incision Lenticule Extraction with Prophylactic Accelerated Collagen Cross-Linking

**DOI:** 10.1155/2022/1640249

**Published:** 2022-07-11

**Authors:** Fei Mo, Yu Di, Ying Li

**Affiliations:** Department of Ophthalmology, Peking Union Medical College Hospital, Chinese Academy of Medical Sciences, Beijing 100730, China

## Abstract

**Purpose:**

To study the corneal morphology and biomechanics in cases of small incision lenticule extraction with prophylactic accelerated collagen cross-linking (SMILE Xtra).

**Methods:**

This study was a retrospective study. 28 eyes of 14 patients with moderate-high risk of postoperative ectasia according to the Randleman scoring system underwent SMILE Xtra procedure. Outcome data were recorded including uncorrected distance visual acuity (UDVA), manifest refraction spherical equivalent (MRSE), surface regularity index (SRI), surface asymmetry index (SAI), simulated keratometry (SimK), posterior axial curvature (PAC), anterior and posterior corneal elevations (ACE and PCE), central corneal thickness (CCT), corneal resistance factor (CRF), corneal hysteresis (CH), and cornea-compensated intraocular pressure (IOPcc). The follow-up period was 12 months.

**Results:**

There were 28, 26, 22, 12, and 10 eyes enrolled at postoperative 1^st^ day and 1^st^, 3^rd^, 6^th^, and 12^th^ months, respectively. The UDVA improved from 1.27 ± 0.18 logMAR preoperatively to -0.06 ± 0.04 logMAR postoperatively (*P* < 0.05). The MRSE improved from -5.05 ± 1.15 *D* preoperatively to -0.14 ± 0.30 *D* postoperatively (*P* < 0.05). SAI, SimK, PAC, PCE, and CCT all changed significantly at 1^st^ month postoperatively (*P* < 0.05) and stabilized during the remainder of the follow-up (*P* > 0.05). There was no significant change in SRI or ACE before and after surgery (*P* > 0.05). CRF, CH, and IOPcc all decreased significantly at 1^st^ month postoperatively (*P* < 0.05) and remained stable afterwards (*P* > 0.05).

**Conclusions:**

The changes in the corneal morphology and biomechanics remained stable after SMILE Xtra, and there was no sign of postoperative ectasia or refractive regression. Combined with the improvement of visual and refractive results, SMILE Xtra may be a promising method for corneal refractive surgeries in patients at risk.

## 1. Introduction

Small incision lenticule extraction (SMILE), a safe and effective procedure for correcting myopia and myopic astigmatism, has been widely accepted globally [1,2]. Theoretically, SMILE may better preserve corneal biomechanical properties than both photorefractive keratectomy (PRK) and laser in-situ keratomileusis (LASIK), as expected given that the strongest anterior lamellae of the cornea remains intact in the SMILE procedure [3]. However, there are still reports of post-SMILE keratectasia, especially in high-risk patients [4].

Corneal collagen cross-linking (CXL) can improve the biomechanical rigidity of the corneal stroma by inducing the formation of intra- and interfibrillar covalent bonds via photosensitized oxidation between riboflavin and ultraviolet A radiation [5–7]. Numerous studies have confirmed CXL's role in halting the progression and promoting corneal stabilization of keratoconus and other forms of ectasia [6,8,9]. In recent years, a simultaneous combination of prophylactic CXL and corneal refractive surgeries (PRK Xtra, LASIK Xtra, and SMILE Xtra) has emerged and shown good visual and refractive results [10–12]. These surgeries aim to improve the cornea's postoperative biomechanical stability and potentially prevent future ectasia in cases where the topographic indices or the clinical history is suggestive of “at risk” corneas. To date, most studies on SMILE Xtra have focused on the visual and refractive outcomes but ignored the corneal morphology and biomechanics changes. Hence, we reported our clinical results on SMILE Xtra, including visual and refractive status, anterior and posterior corneal morphology, and corneal biomechanics.

## 2. Methods

### 2.1. Patients

We employed a retrospective interventional study that included 28 eyes of 14 patients undergoing SMILE Xtra between 2016 and 2017. The study was approved by the Institutional Review Board and adhered to the tenets of the Declaration of Helsinki. There were currently no standardized patient selection criteria for SMILE Xtra. In our refractive surgery center, patients were advised to perform refractive surgery and simultaneous CXL to potentially prevent future ectasia or refractive regression based on any of the following criteria: (1) abnormal preoperative topography including asymmetric bowtie, inferior steepening, skewed radial axis, and a central keratometry value of 48.0D or more; (2) a refractive error of greater than -10.00 diopters (*D*) spherical equivalent; (3) the central corneal thickness of less than 480 *μ*m; (4) the percentage of tissue altered (PTA) of 40% or more; and (5) the changes in the spherical equivalent between 0.50D and 1.00D in recent two years but with special needs for surgery. The patients were adequately informed of the potential risks, benefits, and additional cost and decided to accept the SMILE Xtra procedure or not voluntarily. According to the Randleman scoring system, the enrolled patients were at moderate to high risk of ectasia [13]. Patients aged 18 years and below with predicted residual stromal bed thickness of less than 280 *μ*m, established keratoconus or forme fruste keratoconus, hyperopia or mixed astigmatism, significant ocular diseases, and systemic diseases, those who used medications that would affect the wound healing, and pregnant or lactating mothers were excluded from the study.

### 2.2. Ophthalmic Examinations

All patients included in this study underwent complete ophthalmic examinations pre- and postoperatively. The patients were examined preoperatively and postoperatively on the first day and the first, third, sixth, and twelfth months. There were different rates of loss to follow-up at the postoperative time points.

The following results were assessed and recorded: slit-lamp examination, fundus examination, uncorrected distance visual acuity (UDVA), corrected distance visual acuity (CDVA), and manifest refraction spherical equivalent (MRSE). A-scan ultrasound pachymetry (SP-3000; Tomey) was used to measure the central corneal thickness (A-scan CCT). Corneal topography (TMS-4.3 A; Japan) recorded the topographic pattern, mean keratometry value (Kmean), surface regularity index (SRI), and surface asymmetry index (SAI). The Galilei Dual-Scheimpflug Analyzer (GSA) (Ziemer Ophthalmic Systems AG, Switzerland) was used to measure average simulated keratometry (SimK), posterior axial curvature (PAC), anterior and posterior corneal elevations (ACE and PCE), and central corneal thickness (CCT). The ocular response analyzer (ORA; Reichert Corporation, USA) was used to measure the cornea-compensated intraocular pressure (IOPcc), corneal resistance factor (CRF), and corneal hysteresis (CH).

### 2.3. Surgical Technique

The same surgeon (YL) performed all surgeries. The SMILE procedure was done under topical anesthesia using the VisuMax® 500 kHz femtolaser system (Carl Zeiss Meditec, Jena, Germany). The refractive target was to achieve emmetropia. The following parameters were used: cap thickness, 110–120 *μ*m; cap diameter, 7.0–7.5 mm; lenticule diameter, 6.0–6.5 mm with a transition zone of 0.1 mm; cut energy, 135 nJ; spot distance, 4.5 *μ*m for the lenticule and 2.0 *μ*m for its border; and the side cut incision, 2 mm at the 10 o'clock position of the cornea. After removing the refractive lenticule, 0.22% riboflavin with saline (VibeX Xtra, Avedro) was instilled through the small incision into the corneal pocket and allowed for a soak time of 90 seconds. Then, the photosensitizer was utterly washed out from the pocket using a balanced saline solution. This was followed by an ultraviolet A (UVA) irradiation using the Avedro's (Avedro Inc.) corneal cross-linking system at 30 mW/cm^2^ for 90 seconds (total energy: 2.7 J/cm^2^). No intraoperative complications were recorded.

Postoperative treatments included topical administration of 0.5% levofloxacin (Cravit, Santen) four times a day for two weeks, 0.5% loteprednol etabonate ophthalmic suspension (Lotemax, Bausch & Lomb) four times a day in the first week, followed by tapering dosages for three weeks, and lubricants four times daily for three months.

### 2.4. Statistical Analysis

Data analysis was performed using the SPSS software for MacOS (IBM, version 26.0). Snellen visual acuity values were converted to the logarithm of the minimum angle of resolution (logMAR) units. The quantitative data were described using the mean and standard deviation. A one-way analysis of variance (ANOVA) or the Kruskal–Wallis analysis was used to analyze the data from preoperative to postoperative examinations and between consecutive postoperative visits. The chi-squared test was used to compare the different ratios. Differences were considered statistically significant when the associated *P* value was less than 0.05.

## 3. Results

A total of 28 eyes (14 patients) were included in the preoperation. There were 28, 26, 22, 12, and 10 eyes enrolled at the postoperative first day and first, third, sixth, and twelfth months, respectively. According to the follow-up time, these different numbers of eyes were divided into groups preop/post 1 d, post 1 m, post 3 m, post 6 m, and post 12 m.

All baseline data are summarized in Table 1. Of the 28 eyes, 25 eyes (89.3%) were classified as high risk (score ≥4), while three eyes (10.7%) were classified as moderate risk (a score of 3). In the preoperative period, the mean age was 24.4 years ± 5.24 (SD) (range 18–34 years). The mean CCT measured by the A-scan was 509.96 ± 22.91 *μ*m. The mean spherical equivalent (SE) was −5.05 ± 1.15 D. There were no postoperative complications such as punctate keratitis, deep lamellar keratitis, epithelial ingrowth, ectasia, or regression in any of the eyes throughout the entire follow-up period.

### 3.1. Visual and Refractive Outcomes

**Figure 1:**
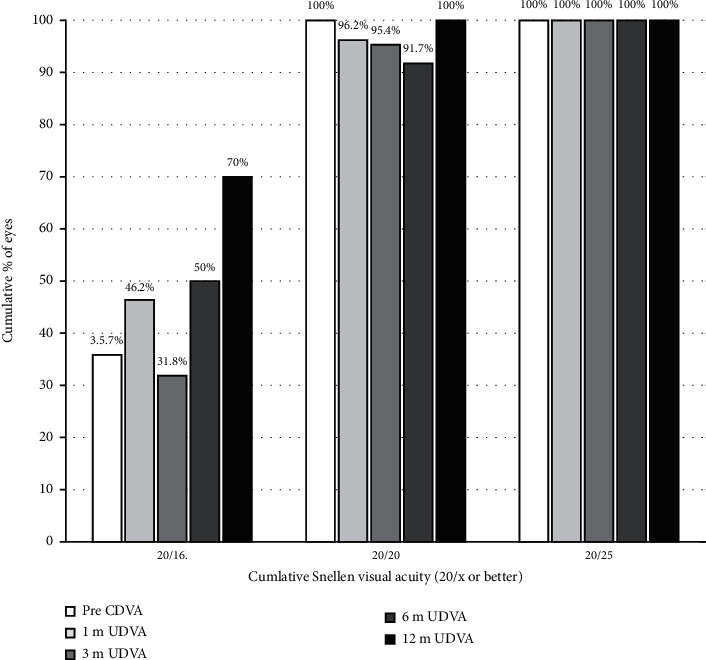
The cumulative percentage of the eyes attaining specified cumulative levels of postoperative uncorrected distance visual acuity (UDVA).

**Figure 2:**
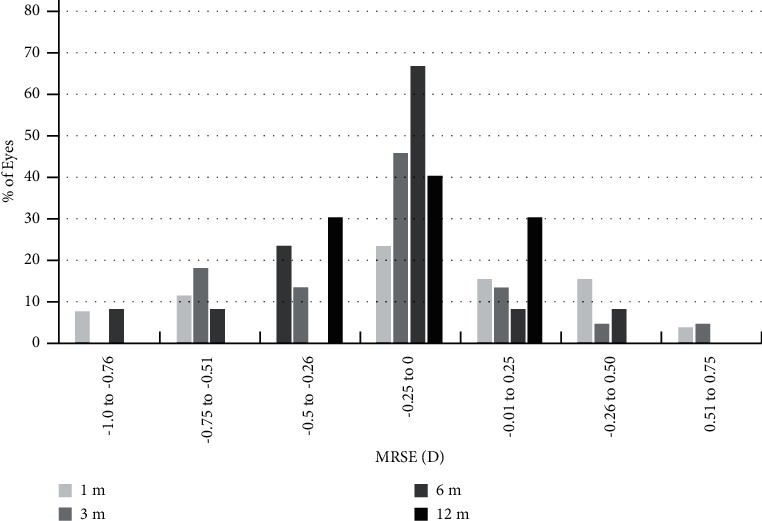
The distribution of postoperative manifest refraction spherical equivalent (MRSE).

Changes in the visual acuity and refraction over time are shown in Tables 2 and 3. There was a significant improvement in logMAR UDVA on the first day and up to the first month after surgery (*P*=0.017, *P* < 0.001, respectively), with no significant changes afterwards (*P* > 0.05). The MRSE had a remarkable reduction after the surgery when contrasted to the preoperation (*P* < 0.001), with no significant changes during the remainder follow-up (*P* > 0.05). At 1, 3, 6, and 12 months postoperatively, a UDVA of 20/20 or better was achieved for 96.2%, 95.4%, 91.7%, and 100% of the individuals, respectively (Figure 1). At postoperative 1, 3, 6, and 12 months, 77%, 77.3%, 83.4%, and 100% of eyes were within ±0.50D from target, respectively. All eyes (100%) were within ±1.00D at any postoperative time points (Figure 2).

### 3.2. Corneal Morphology

The parameters of corneal morphology included Kmean, SRI, SAI, anterior and posterior corneal curvature (SimK and PAC), anterior and posterior corneal elevations (ACE and PCE), and CCT. Changes in the corneal morphology during the follow-up period are shown in Tables 2 and 3. The Kmean and SimK showed similar changes. They decreased significantly one month after surgery and then increased gradually, although the latter change was not statistically significant (Kmean: *P* < 0.001, >0.05; SimK: *P* < 0.001, >0.05). There was no significant change in SRI or ACE before and after surgery (SRI: *P*=0.556; ACE: *P*=0.677). The SAI significantly increased at one month postoperation compared with the preoperation and remained stable over the subsequent postoperative follow-up (*P*=0.003, >0.05). For the posterior corneal surface, both PAC (absolute value) and PCE increased significantly at postoperative one month (PAC: *P*=0.006; PCE: *P*=0.023). They reached the maximum at the third month postoperatively although the difference between the consecutive postoperative follow-up was not significant (PAC: *P* > 0.05; PCE: *P* > 0.05). The CCT decreased markedly at one month postoperatively (*P* < 0.001). Similarly, the CCT also reached the minimum at three months postoperatively, but with no statistical significance during the whole postoperative follow-up (*P* > 0.05).

### 3.3. Corneal Biomechanics

Both CRF and CH decreased significantly at postoperative one month and remained stable during the 12 months of follow-up (CRF: *P* < 0.001, >0.05; CH: *P* < 0.001, >0.05) (Tables 2 and 3). The IOPcc had a remarkable reduction at one month postoperatively (*P*=0.025). The IOPcc also reached the minimum at three months postoperatively, but with no statistical significance during the postoperative follow-up (*P* > 0.05).

## 4. Discussion

Postoperative keratectasia is one of the most feared complications in corneal refractive surgery. Any corneal refractive surgery will undoubtedly destroy the biomechanical stability of the cornea. Therefore, the use of simultaneous CXL to restrengthen or compensate for the corneal biomechanics has been proposed. A few studies have reported the favorable safety, efficacy, predictability, and stability of the SMILE Xtra procedure in patients at risk of postectasia [12,14,15]. However, they lacked direct evidence of the corneal morphology and biomechanics to evaluate the actual cornea status. Only one study has measured the corneal biomechanics. But it did not include corneal hysteresis, which was another important biomechanical parameter in addition to the corneal resistance factor [16]. A previous study by our team has compared the corneal morphology and biomechanical properties between SMILE Xtra and LASIK Xtra. However, the follow-up of the study was intermittent, resulting in limitations in showing the postoperative changes [17]. Therefore, we reported the anterior and posterior corneal morphology and complete biomechanical properties on SMILE Xtra through a continuous 12-month follow-up.

There were promising results on the SMILE Xtra regarding the visual and refractive outcomes. At the 12-month visit, there was a slight myopic shifting trend of MRSE (−0.14 ± 0.30D), and the percentage of eyes with UDVA of 20/20 or better was 100%. This result was comparable to the results reported by Ganesh et al., which exhibited −0.24 ± 0.18D of MRSE and 95% UDVA [14].

Regarding the corneal morphology, we found good stability after surgery in corneal curvatures and elevations, either on the anterior or posterior surfaces. For the anterior corneal curvature, Ganesh et al. showed stable keratometry over 12-month time [14]. In this study, both Kmean and SimK showed a slowly rising trend from the third month postoperatively, but with no statistical significance. The difference in K reading changes between the two studies may be related to their different CXL protocols and total energies, resulting in different flattening effects on the cornea. We found the stable SRI and varying SAI before and after surgery for the corneal surface regularity and asymmetry. The SAI that increased after surgery may be related to the inconsistent activity of the corneal stromal healing reaction and the collagen cross-linking reaction in local parts. It is well known that the first sign of keratectasia appears earlier on the posterior corneal surface than on the anterior [18]. Therefore, changes in the posterior surface parameters played a more critical role in predicting the occurrence of keratectasia. The posterior corneal surface elevation is a highly sensitive and specific indicator for discriminating corneal ectasia [19, 20]. Konstantopoulos et al. found that the maximum posterior elevation (MPE) increased significantly at week six after SMILE Xtra in a rabbit model [21]. In the present study, the PCE had a statistical increase at one month and peaked at 3 months postoperatively, gradually dropping. Interestingly, there were similar changes in PAC, CCT, and IOPcc, exhibiting the most noticeable difference between pre- and postoperation in the third month. This phenomenon suggests a slight forward displacement of the posterior corneal surface with corneal thickness thinning. The change in CCT was in line with that reported in Osman et al., who reported a statistically significant decrease from one to three months, which increased again after six months and was attributed to the compaction of the corneal stroma because of CXL [16]. A study based on in vivo confocal laser microscopy observed that typical honeycomb-like corneal edema was found on the surgical interface at one month after SMILE Xtra and disappeared gradually [22]. So, we speculated that dehydration may also account for further reduction of the CCT from three months to one month postoperatively. Kohnen et al. proposed corneal thickening in the postoperative follow-up, and the corneal thickness was significantly higher after 12 months compared to one month after FS-LASIK Xtra [23]. Similarly, we also found that the CCT reached its thickest at the twelfth month, although the difference between the postoperative follow-up was not significant. The following reasons may account for sustained corneal thickness growth after surgery. First, the stromal changes from lenticule extraction and CXL could result in consistent corneal epithelial hyperplasia and remodeling so as to uphold the integrity of the optical surface of the cornea [24, 25]. Second, the increased reflectivity and density of the extracellular matrix stroma after SMILE Xtra indicated corneal stroma modification [22, 26]. Correspondingly, the forward displacement of the posterior corneal surface gradually improved with the thickening of the cornea, especially in the posterior corneal elevation.

Corneal biomechanics is the characteristic of the deformation and equilibrium of the corneal tissue under the application of any force [27]. The CRF appears to be an indicator of the overall resistance of the cornea, whereas the CH represents the ability of corneal tissue to absorb and dissipate energy [28]. A previous study reported that CRF was significantly higher in the SMILE Xtra group compared to the SMILE-only group and remained stable over a 24-month follow-up [16]. However, the lack of measurements of CH was a disadvantage in this reported study. In the present research, we found that both the CRF and CH were significantly reduced after surgery but remained stable over the 12 months of follow-up. This significant decrease after surgery could be interpreted as being a result of the SMILE procedure itself [29]. In addition, the stable features of the CRF and CH during the postoperative follow-up in such a high proportion of moderate-high risk patients indicated that prophylactically simultaneous use of CXL with SMILE may have the potential to prevent postectasia for patients at risk.

There is currently no consensus on patient selection criteria for refractive surgery and simultaneous CXL. In addition to the inclusion criteria adopted by our refractive surgery center, all enrolled eyes were also assessed by the Randleman scoring system, and the ectasia risk was moderate to high. Although this scoring system was developed for LASIK, it was also applied here given that a modified version does not yet exist for SMILE. Moreover, we believed that the risk factors for ectasia should be applied uniformly to both procedures although the biomechanical superiority of SMILE over LASIK is attributed to the stromal tissue removal at the deeper layers [30]. Hence, the results of the Randleman scoring system may be used as a supplementary reference for the ectasia risk after SMILE, and moderate-to-high risk patients should be treated with caution.

Currently, there are no standardized protocols for prophylactic CXL with refractive surgery. Existing studies on SMILE Xtra have reported different irradiation protocols, where the total energy dose of the UVA ranges from 0.8 to 5.4 J/cm^2^ [12, 14, 31, 32]. Too little energy would be insufficient to achieve the desired effect. At the same time, too much energy may cause haze and a continued corneal flattening effect [33]. According to the Bunsen–Roscoe law of reciprocity, the photochemical biological impact of the ultraviolet light is proportional to the total energy dose delivered, regardless of the applied irradiance and time [34]. Thus, to avoid the potential risks of a long period of corneal exposure, accelerated CXL, the same photochemical effect with reducing illumination time and correspondingly increasing irradiation intensity, would be a better option. In our study, we soaked the intrastromal pocket with riboflavin solution for 90 seconds and adopted the high influence of the Averdo KXL device at 30 mW/cm^2^ and accelerated CXL for 90 seconds, thus delivering a total energy of 2.7 J/cm^2^(0.03 W/cm^2^ × 90 seconds = 2.7 J/cm^2^). This protocol proved safe and effective, as all eyes were exempted from complications. We did not routinely check the endothelial cell count as it has already been shown that no variation in cell count or the endothelial mosaic was observed after SMILE Xtra [22].

The advantage of this study was that we evaluated the changes in the corneal morphology on both the anterior and posterior surfaces and completed corneal biomechanics before and after SMILE Xtra. In addition, the follow-up time was relatively long and continuous. Unfortunately, the sample size was small, and the study was retrospective. Another limitation of this study was that we did not compare the outcomes with SMILE alone. A prospective study comparing SMILE Xtra with SMILE alone in a larger sample size and longer duration of follow-up might be required in the future.

## 5. Conclusions

In conclusion, the changes in the corneal morphology and biomechanics remained stable after SMILE Xtra, and there was no sign of postoperative ectasia or refractive regression. Combined with the improvement of visual and refractive results, SMILE Xtra may be a promising method for corneal refractive surgeries in patients at risk.

## Figures and Tables

**Table 1:** Preoperative baseline data of patients who underwent SMILE Xtra.

Preop. parameters	Groups						
Mean ± SD	Preop/post 1 d (28 eyes)	Post 1 m (26 eyes)	Post 3 m (22 eyes)	Post 6 m (12 eyes)	Post 12 m (10 eyes)	F value	*P* value
Male: female	6/8	5/8	5/6	3/3	3/2	0.996	0.966^a^<>(2-sided)
Age (years)	24.36 ± 5.24	24.54 ± 5.41	24.91 ± 5.49	25.00 ± 6.99	24.40 ± 6.19	0.024	0.999^b^
UDVA (logMAR)	1.27 ± 0.18	1.29 ± 0.17	1.31 ± 0.15	1.26 ± 0.12	1.25 ± 0.14	0.448	0.773^b^
MRSE (D)	−5.05 ± 1.15	−5.23 ± 0.99	−5.38 ± 1.00	−4.68 ± 1.08	−4.28 ± 1.40	2.247	0.070^b^
A-scan CCT(*μ*m)	509.96 ± 22.91	512.50 ± 21.76	509.45 ± 21.66	505.33 ± 24.66	498.6 ± 18.79	0.807	0.523^b^
Kmean	43.84 ± 1.40	44.00 ± 1.30	44.06 ± 1.40	44.34 ± 1.79	43.85 ± 2.29	0.263	0.901^b^
SRI	0.20 ± 0.19	0.22 ± 0.20	0.20 ± 0.18	0.29 ± 0.25	0.17 ± 0.18	0.592	0.669^b^
SAI	0.30 ± 0.13	0.30 ± 0.13	0.28 ± 0.13	0.34 ± 0.17	0.34 ± 0.18	0.498	0.737^b^
SimK (D)	43.90 ± 1.37	44.06 ± 1.28	44.10 ± 1.38	44.40 ± 1.76	43.90 ± 2.23	0.266	0.899^b^
PAC (D)	−6.34 ± 0.24	−6.37 ± 0.23	−6.36 ± 0.25	−6.41 ± 0.28	−6.35 ± 0.36	0.169	0.954^b^
ACE (*μ*m)	3.18 ± 1.02	3.15 ± 1.05	3.00 ± 0.93	3.25 ± 1.29	3.20 ± 1.03	0.151	0.962^b^
PCE (*μ*m)	7.71 ± 5.00	8.04 ± 5.03	7.91 ± 5.24	9.17 ± 7.08	8.50 ± 7.74	0.159	0.959^b^
CCT (*μ*m)	532.07 ± 21.45	534.38 ± 20.46	531.55 ± 19.53	528.00 ± 23.48	521.00 ± 14.18	0.866	0.488^b^
CRF	9.51 ± 1.37	9.62 ± 1.34	9.42 ± 1.28	9.39 ± 1.67	8.68 ± 1.42	0.867	0.487^b^
CH	9.98 ± 1.49	10.07 ± 1.49	9.76 ± 1.32	9.81 ± 1.89	9.05 ± 1.41	0.921	0.455^b^
IOPcc (mmHg)	14.89 ± 2.62	14.88 ± 2.71	15.42 ± 2.57	15.13 ± 2.63	15.71 ± 2.27	0.313	0.869^b^

UDVA: uncorrected distance visual acuity; MRSE: manifest refraction spherical equivalent; A-scan CCT: central corneal thickness measured by A-scan ultrasound pachymetry; Kmean: mean keratometry value; SRI: surface regularity index; SAI: surface asymmetry index; SimK: simulated keratometry; PAC: posterior axial curvature; ACE: anterior corneal elevations; PCE: posterior corneal elevations; CCT: central corneal thickness measured by the Galilei Dual-Scheimpflug Analyzer; CRF: corneal resistance factor; CH: corneal hysteresis; IOPcc: cornea-compensated intraocular pressure. ^a^Fisher's exact test; ^b^one-way analysis of variance (ANOVA).

**Table 2:** Changes in visual acuity, refraction, corneal morphology, and corneal biomechanics over time.

Parameters Mean ± SD	Preop	Post 1 d	Post 1 m	Post 3 m	Post 6 m	Post 12 m	F value	*P* value
UDVA (logMAR)	1.27 ± 0.18	0.13 ± 0.13	−0.04 ± 0.05	−0.02 ± 0.04	−0.04 ± 0.05	−0.06 ± 0.04	100.761	<0.001
MRSE (D)	−5.05 ± 1.15	−0.78 ± 0.64	−0.18 ± 0.49	−0.16 ± 0.39	−0.15 ± 0.37	−0.14 ± 0.30	76.821	<0.001
Kmean (D)	43.83 ± 1.40	—	39.56 ± 1.73	39.83 ± 2.00	40.39 ± 2.38	40.61 ± 2.62	21.753	<0.001
SRI	0.20 ± 0.19	—	0.26 ± 0.19	0.24 ± 0.16	0.21 ± 0.17	0.15 ± 0.19	0.758	0.556
SAI	0.30 ± 0.13	—	0.58 ± 0.45	0.52 ± 0.18	0.60 ± 0.24	0.60 ± 0.16	5.125	0.001
SimK (D)	43.90 ± 1.37	—	39.31 ± 1.83	39.54 ± 2.15	40.09 ± 2.58	40.40 ± 2.65	23.365	<0.001
PAC (D)	-6.34 ± 0.24	—	-6.60 ± 0.24	-6.69 ± 0.30	−6.60 ± 0.25	-6.62 ± 0.30	6.538	<0.001
ACE (*μ*m)	3.18 ± 1.02	—	3.62 ± 2.02	3.00 ± 1.80	3.33 ± 2.31	4.20 ± 2.44	2.32	0.677
PCE (*μ*m)	7.71 ± 5.00	—	12.19 ± 6.90	15.45 ± 8.96	10.08 ± 3.58	9.50 ± 4.45	16.446	0.002
CCT (*μ*m)	532.07 ± 21.45	—	435.15 ± 25.56	431.00 ± 27.21	446.33 ± 22.87	453.00 ± 18.54	78.83	<0.001
CRF (mmHg)	9.51 ± 1.37	—	6.07 ± 0.94	6.21 ± 0.62	6.10 ± 0.36	6.32 ± 0.60	54.943	<0.001
CH (mmHg)	9.98 ± 1.49	—	7.68 ± 0.89	7.86 ± 0.68	7.50 ± 0.74	7.56 ± 0.69	42.393	<0.001
IOPcc (mmHg)	14.89 ± 2.62	—	13.10 ± 2.22	12.90 ± 1.56	13.78 ± 1.82	14.19 ± 1.52	3.632	0.009

UDVA: uncorrected distance visual acuity; MRSE: manifest refraction spherical equivalent; Kmean: mean keratometry value; SRI: surface regularity index; SAI: surface asymmetry index; SimK: simulated keratometry; PAC: posterior axial curvature; ACE: anterior corneal elevations; PCE: posterior corneal elevations; CCT: central corneal thickness measured by the Galilei Dual-Scheimpflug Analyzer; CRF: corneal resistance factor; CH: corneal hysteresis; IOPcc: cornea-compensated intraocular pressure.

**Table 3:** The differences at 1, 3, 6, and 12 months after SMILE Xtra surgery.

	UDVA					MRSE						
	Preop	1 d	1 m	3 m	6 m	Preop	1 d	1 m	3 m	6 m		
1 d	0.017					<0.001						
1 m	<0.001	<0.001				<0.001	0.112					
3 m	<0.001	0.001	1.000			<0.001	0.139	1.000				
6 m	<0.001	0.004	1.000	1.000		<0.001	0.277	1.000	1.000			
12 m												
	<0.001	0.001	1.000	1.000	1.000	<0.001	0.385	1.000	1.000	1.000		

	*Kmean*				*SAI*				*simK*			
	Preop	1 m	3 m	6 m	Preop	1 m	3 m	6 m	Preop	1 m	3 m	6 m
1 m	<0.001				0.003				<0.001			
3 m	<0.001	1.000			0.062	1.000			<0.001	1.000		
6 m	<0.001	1.000	1.000		0.021	1.000	1.000		<0.001	1.000	1.000	
12 m	<0.001	1.000	1.000	1.000	0.030	1.000	1.000	1.000	<0.001	1.000	1.000	1.000

	*PAC*				*PCE*				*CCT*			
	Preop	1 m	3 m	6 m	Preop	1 m	3 m	6 m	Preop	1 m	3 m	6 m
1 m	0.006				0.023				<0.001			
3 m	<0.001	1.000			0.002	1.000			<0.001	1.000		
6 m	0.059	1.000	1.000		0.728	1.000	1.000		<0.001	1.000	0.771	
12 m	0.046	1.000	1.000	1.000	1.000	1.000	0.980	1.000	<0.001	0.477	0.178	1.000

	*CRF*				*CH*				*IOPcc*			
	Preop	1 m	3 m	6 m	Preop	1 m	3 m	6 m	Preop	1 m	3 m	6 m
1 m	<0.001				<0.001				0.025			
3 m	<0.001	1.000			<0.001	1.000			0.014	1.000		
6 m	<0.001	1.000	1.000		<0.001	1.000	1.000		1.000	1.000	1.000	
12 m	<0.001	1.000	1.000	1.000	<0.001	1.000	1.000	1.000	1.000	1.000	1.000	1.000

UDVA: uncorrected distance visual acuity; MRSE: manifest refraction spherical equivalent; Kmean: mean keratometry value; SRI: surface regularity index; SAI: surface asymmetry index; SimK: simulated keratometry; PAC: posterior axial curvature; ACE: anterior corneal elevations; PCE: posterior corneal elevations; CCT: central corneal thickness measured by the Galilei Dual-Scheimpflug Analyzer; CRF: corneal resistance factor; CH: corneal hysteresis; IOPcc: cornea-compensated intraocular pressure.

## Data Availability

The datasets obtained and/or analyzed during the current study are available from the corresponding author on reasonable request.
